# A mutation in negative regulator of basal resistance *WRKY17 *of *Arabidopsis* increases susceptibility to *Agrobacterium*-mediated genetic transformation

**DOI:** 10.12688/f1000research.2-33.v1

**Published:** 2013-02-06

**Authors:** Benoît Lacroix, Vitaly Citovsky

**Affiliations:** 1 Department of Biochemistry and Cell Biology, State University of New York, New York, 11794-5215, USA

**Keywords:** WRKY17, Arabidopsis, Agrobacterium

## Abstract

*Agrobacterium* is a phytopathogenic bacterium that induces crown gall disease in many plant species by transferring and integrating a segment of its own DNA (T-DNA) into its host genome. Whereas *Agrobacterium* usually does not trigger an extensive defense response in its host plants, it induces the expression of several defense-related genes and activates plant stress reactions. In the complex interplay between *Agrobacterium* and its host plant, *Agrobacterium* has evolved to take advantage of these plant defense pathways for its own purpose of advancement of the infection process. For example, *Agrobacterium* utilizes the host stress response transcriptional regulator VIP1 to facilitate nuclear import and proteasomal uncoating of its T-DNA during genetic transformation of the host cell. In *Arabidopsis*, the *VIP1* gene expression is repressed by WRKY17, a negative regulator of basal resistance to *Pseudomonas*. Thus, we examined whether WRKY17 is also involved in plant susceptibility to genetic transformation by *Agrobacterium*. Using reverse genetics, we showed that a *wrky17* mutant displays higher expression of the *VIP1 *gene in roots, but not in shoots. In a root infection assay, the *wrky17 *mutant plants were hyper-susceptible to *Agrobacterium* compared to wild type plants. WRKY17, therefore, may act as a positive regulator of *Arabidopsis* resistance to *Agrobacterium*. This notion is important for understanding the complex regulation of *Agrobacterium*-mediated genetic transformation; thus, although this paper reports a relatively small set of data that we do not plan to pursue further in our lab, we believe it might be useful for the broad community of plant pathologists and plant biotechnologists.

## Introduction

The WRKY protein family is composed of at least 74 members in *Arabidopsis thaliana* ^1^; they act as transcriptional regulators and participate mainly in the control of gene expression involved in the plant stress response, and, particularly, in the induction of gene expression by pathogen-derived elicitors. *Arabidopsis* WRKY17, together with another family member WRKY11, is a negative regulator of the basal defense response ^2^. The *wrky17* and *wrky11* genes are usually induced during the defense response, and *Arabidopsis* loss-of-function mutants *wrky17* and *wrky11* display higher expression of numerous stress- or defense-related genes and show increased resistance to infection by *Pseudomonas*, but not by other pathogens. Thus, *wrky17* and *wrky11* have been suggested to play a role in the fine-tuning of the defense response, avoiding the effect of excessive reaction ^2^.

Among the target genes of wrky17/wrky11 is *vip1*, which is overexpressed in both *wrky*11 and *wrky*17 mutants ^2^. VIP1 is a multifunctional bZIP transcription factor that stimulates stress- and defense-related gene expression by binding to a specific DNA hexamer motif present in many promoters that respond to activation of the MPK3 pathway ^3^, including the *PR1* pathogenesis-related gene ^4^. VIP1 might also be involved in other stress-dependent regulation pathways, such as osmosensory signaling ^5^. Interestingly, the VIP1-related defense responses are activated during *Agrobacterium*-host plant interactions, and *Agrobacterium* has evolved to subvert them to facilitate the infection process ^4, 6^.

VIP1, a host protein initially discovered as an interacting partner of the *Agrobacterium* T-DNA packaging protein VirE2 ^7^, is involved in several critical aspects of plant genetic transformation by *Agrobacterium*. Specifically, VIP1 is thought to facilitate nuclear import of the T-DNA-protein complexes ^7– 9^, their targeting to the host chromatin ^10– 12^, and proteasomal uncoating of the T-DNA molecule from its associated proteins prior to integration ^13– 15^. Thus, we investigated one of the VIP1-controlling *WRKY* mutants, *wrky17*, in regard to *vip1* expression and the potential effects on *Agrobacterium* infection.

## Results and discussion

### *VIP1* represents one of the target genes of *WRKY17*

A previous microarray analysis of the *wrky17* mutant identified a number of upregulated genes ^2^, one of which, *VIP1*, represents a major player in plant genetic transformation by *Agrobacterium* ^7, 10, 13^. However, microarray analyses of gene expression, although commonly used, often yield divergent data ^16, 17^ and, therefore, require direct confirmation by detection of the specific transcripts. Thus, we analyzed the *wrky17* mutant for the levels of *VIP1* expression.

**Figure 1.**
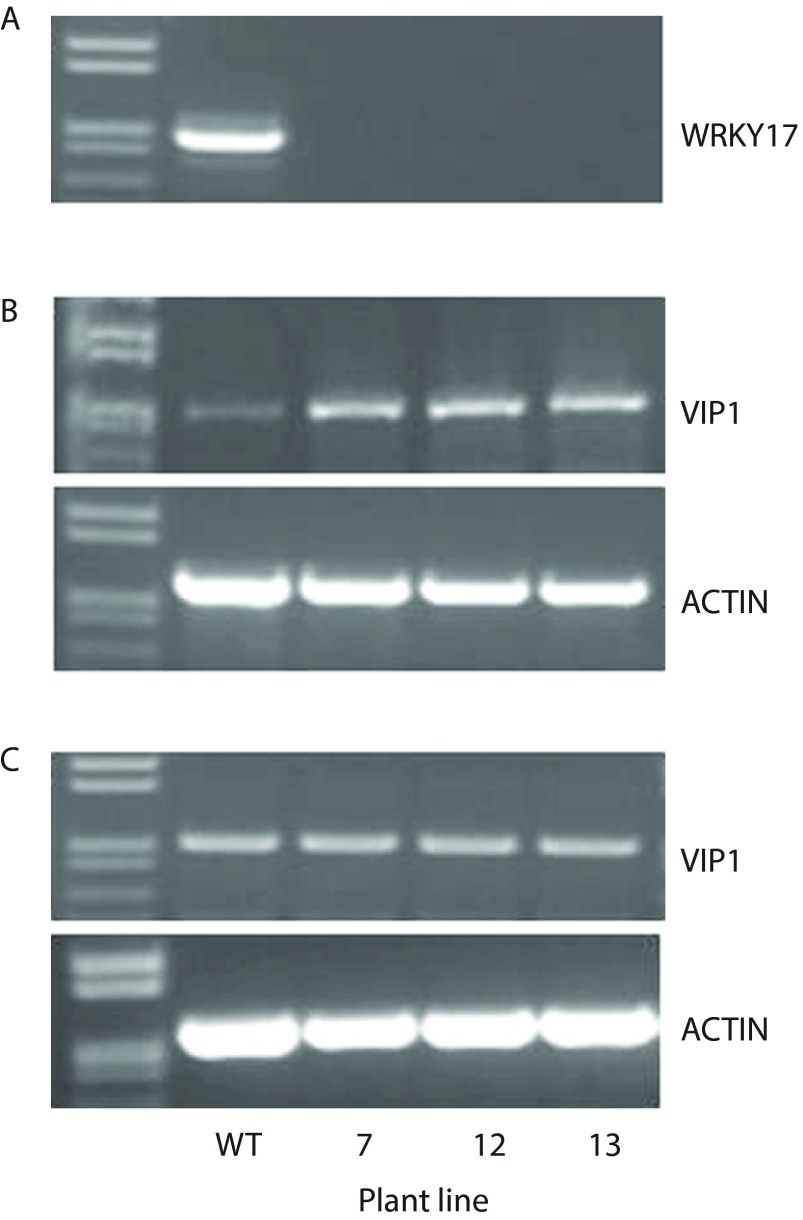
RT-PCR analysis of *ACTIN2*, *WRKY17* and *VIP1* gene expression in wild-type and *wrky17* mutant *Arabidopsis* plants. ( **A**) *WRKY17* expression in whole plants. ( **B**, **C**) *VIP1* expression in roots and shoots, respectively. WT, wild-type plants; 7, 12, and 13 are the three different lines of the homozygous *wrky17-1* mutant.

First, we examined three different lines of *Arabidopsis* plants derived from the *wrky17-1* mutant ^2^ for the presence of the *WRKY17* transcript using RT-PCR. Figure 1A shows that whereas the wild-type plants produced *WRKY17* mRNA, neither of the mutant lines accumulated detectible levels of this transcript. Next, we investigated the effect of the *wrky17* mutation on the expression of the *VIP1* gene. Using RT-PCR, we analyzed the levels of the *VIP1* transcript in plant roots ( Figure 1B) and shoots ( Figure 1C). The *VIP1* transcription activity was substantially higher in the roots of all three *wrky17* mutants than in those of wild type plants ( Figure 1B). Unexpectedly, we detected no changes in *VIP1* expression in the shoots of the same plants, which accumulated *VIP1* transcripts in amounts similar to those in the wild-type plants ( Figure 1C). Analysis of *ACTin2*-specific transcripts detected similar amounts of PCR products in all samples, indicating equal efficiencies of the RT-PCR reactions ( Figure 1B, C). Collectively, these data suggest that WRKY17 represents one of the transcriptional regulators of the *VIP1* gene, but that this regulation is tissue-specific.

This is consistent with the previous observations of differential regulation of *VIP1* expression during plant development as well as in response to various stimuli. For example, *VIP1* transcription is activated upon induction of cell division ^18^, after osmotic stress, and is differentially expressed in different tissues of *Arabidopsis* ^5^. WRKY17 functions as a transcription inhibitor of several genes involved in plant defense pathways ^1^. Our results suggest that *VIP1* is one of the target genes down-regulated, directly or indirectly, by WRKY17 in tissue-specific fashion. Alternatively, *VIP1* expression in the shoot tissue could be regulated by additional factors which mask the effect of the *WRKY17* knock-out mutation.

### The *wrky17* mutant is hypersusceptible to *Agrobacterium-mediated* genetic transformation

**Figure 2.**
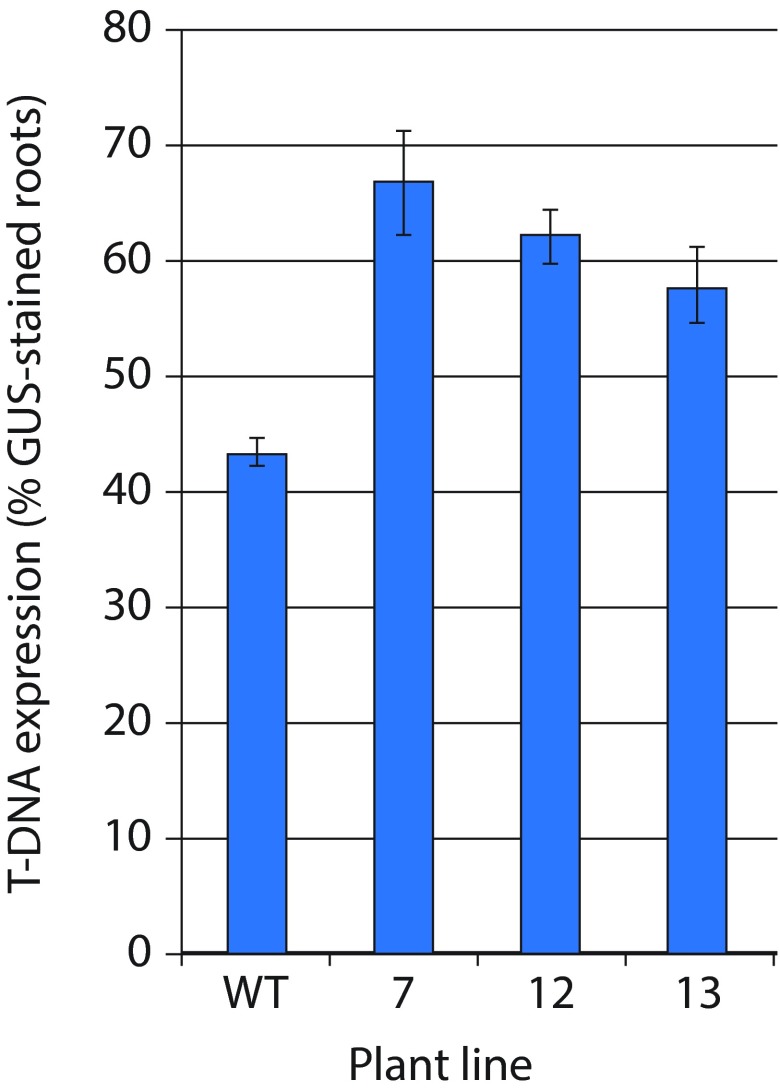
The effect of *wrky17* mutation on susceptibility of *Arabidopsis* roots to *Agrobacterium* infection. Transformation efficiency is expressed as the percent of GUS-stained roots from the total number of roots tested. All data represent average values of three independent experiments with indicated standard deviations. WT, wild-type plants; 7, 12, and 13 are the three different lines of the homozygous *wrky17-1* mutant.

Once we had identified plant tissue showing a clear effect of WRKY17 on *VIP1* expression, we investigated whether this effect altered susceptibility to *Agrobacterium* infection. To this end, we employed the classical *Arabidopsis* root infection assay ^19^, in which the efficiency of infection is monitored and quantified by measuring the level of transient T-DNA expression, that is early expression of the invading T-DNA molecules before their stable integration in the host genome. Root segments from the wild-type and *wrky17* plants were inoculated with *Agrobacterium* strain EHA105 harboring the binary plasmid pBISN1 with the β-glucuronidase (GUS) gene expression reporter in its T-DNA region. T-DNA expression was quantified based on the percentage of root segments exhibiting GUS histochemical staining. These experiments revealed that T-DNA expression frequencies in roots of all three *wrky17* mutant lines were 30–50% higher than those measured in roots of the wild-type plants ( Table 1 and Figure 2).

**Table 1.** Number of root segments staining positive for β-glucuronidase (GUS). Percentage (number of GUS positive root segments/total number of root segments).

Line	Experiment 1	Experiment 2	Experiment 3	Average
WT	43.1% (53/123)	44.7% (68/152)	42.7% (56/131)	43.5%
7	69.2% (72/104)	61.5% (88/143)	70.2% (80/114)	67.0%
12	59.5% (97/163)	63.5% (61/96)	63.8% (81/127)	62.3%
13	60.8% (79/130)	54.1% (72/133)	59.4% (60/101)	58.1%

The increased susceptibility of the *wrky17* roots to *Agrobacterium* infection correlates with elevated transcription levels of the *VIP1* gene in this tissue. Considering the known role of VIP1 as an enhancer of *Agrobacterium* infectivity ^7– 15^, it is likely that higher VIP1 expression in roots of the *wrky17* mutant is responsible for the increased susceptibility to *Agrobacterium*. This notion is consistent with our earlier observations that overexpression of VIP1 in tobacco further elevates transformation efficiency ^8^. That we detected this effect of the *wrky17* mutation using a transient T-DNA expression assay indicates that increased *VIP1* expression affects the early steps of the infection process, i.e., those that occur prior to T-DNA integration and stable expression.

## Conclusion

We show here that the *wrky17* mutant displays elevated *VIP1* expression in its roots as well as increased susceptibility to *Agrobacterium*-induced genetic transformation. This correlation allows a new insight into the interactions between *Agrobacterium* and its host plants. Specifically, this interaction appears to be affected negatively by WRKY17 such that the infection process is enhanced in the loss-of-function *wrky17* mutant. Thus, WRKY17 may represent one of the host factors that elevate resistance to *Agrobacterium* infection in different plant species and tissues that may vary widely in their susceptibility to *Agrobacterium* ^20, 21^. This is unlike the known role of WRKY17 as a negative regulator of plant resistance to *Pseudomonas* ^2^. Although this paper reports a relatively small set of data that we do not plan to pursue further in our lab, we believe its publication will be useful for the broad community of plant pathologists and plant biotechnologists.

## Materials and methods

### Transgenic plants

*Arabidopsis thaliana* plants, wild-type (ecotype Col0) or *wrky17-1* T-DNA insertion mutants (obtained from D. Roby, CNRS Montpellier, France), were grown either in soil or on Gamborg’s B5 medium (20 g.L ^-1^ sucrose, 8 g.L ^-1^ agar), after seed surface sterilization. All plants were grown in an environment-controlled growth chamber at 22°C under long day (16h light/8h dark) conditions. Three lanes of homozygous plants (lanes 7, 12, 13) were isolated from the original *wrky17-1* stock.

### RT-PCR

Total RNA was extracted from plant tissues using Trizol (Invitrogen), and cDNA synthesis was performed with a RevertAid cDNA synthesis Kit (Fermentas) according to the manufacturer’s instructions. Transcript levels were then estimated by PCR, with 30 cycles of amplification. The resulting cDNA was PCR-amplified for 30 cycles using primers specific for the tested gene or for *ACTIN2* as an internal control of a constitutively expressed gene. The following primer pairs were used: 5´ATGACCGTTGATATTATGCGTTTAC3´/5´TCAAGCCGAACCAAACACCAAAC3´ that amplify the full length 966-bp *WRKY17* (At2g24570) cDNA, 5´ATGGAAGGAGGAGGAAGAGG3´/5´TCAGCCTCTCTTGGTGAAATCC3´ that amplify the full length 1,026-bp *VIP1* cDNA, and 5´ATGGCTGAGGCTGATGATATT3´/5´TTAGAAACATTTTCTGTGAACGATTCC3´ that amplify the full length 1,134 bp *ACTIN2* (At3g18780) cDNA.

### Root transformation assay

All infection assays were performed as described by Gelvin (2006) ^19^ with the *Agrobacterium tumefaciens* strain EHA105 (from S. Gelvin, Purdue University, USA), harboring a pBISN1 binary plasmid with an intron-containing *GUS* reporter gene that is not expressed in bacteria ^22^. One-cm long root segments were excised from 3–4 week-old *Arabidopsis* plants grown on Gamborg’s B5 medium, and bundles of root segments were placed on the MS (Murashige and Skoog) medium. For each experiment, roots were pooled from more than 20 plants and divided into three bundles, each containing more than 100 root segments. Root bundles were overlaid with EHA105 harboring pBISN1 suspension culture at A _600_ = 0.25 in NaCl 0.9%, and excess liquid was removed by pipette aspiration after 15 min of incubation. Root segments were then incubated for two days at 22°C under the long day conditions, rinsed in water containing 100 mg.L ^-1^ timentine (BioWorld) to eliminate bacteria, and incubated for an additional three days on the MS medium supplemented with timentine. Root segments were then subjected to the GUS histochemical assay ^23^, with overnight incubation at 37°C, and the number of root segments displaying GUS staining was recorded.
