# Supplementary material for: A mutation in negative regulator of basal resistance WRKY17 of Arabidopsis increases susceptibility to Agrobacterium-mediated genetic transformation
Source: F1000Res. 2013 Feb 6;2:33. [Version 1] doi: 10.12688/f1000research.2-33.v1 (PMC3799546; doi:10.12688/f1000research.2-33.v1)
Supplement: Peer review history

# Peer review: Referee response for version 1

**DOI:** 10.5256/f1000research.852.r759

**Published:** 2013-02-08

**Authors:** Kiran Mysore

**Affiliations:** 1 Plant Biology Division, The Samuel Roberts Noble Foundation, Ardmore, Oklahoma, USA

This is a very short report regarding a finding that may be important for some researchers. Even though the science is acceptable several things need to be changed. Genetic transformation normally refers to stable transformation. The authors have only looked at transient transformation which may or may not be stable (they could have tested this). Therefore, please change the title and the abstract to indicate transient transformation rather than just saying genetic transformation. 

All wild-type gene names should be in capital (e.g., *WRKY17*, *WRKY 11*, *VIP1* etc,.). Only the mutants should be in small letters (e.g., *wrky17*).

The authors mention that the VIP1 gene is induced substantially in the *wrky17* mutant. However, to my eyes the induction is subtle (probably 2-3 fold). They could have done a better quantification using real-time RT-PCR. Please remove the word “substantial”.

Please give the concentration of the *Agrobacterium* used for infection in CFU.

I believe the antibiotic used should be “timetin” and not  “timentine”

I have read this submission. I believe that I have an appropriate level of expertise to confirm that it is of an acceptable scientific standard.

# Peer review: Referee response for version 1

**DOI:** 10.5256/f1000research.852.r754

**Published:** 2013-02-07

**Authors:** Herman Scholthof

**Affiliations:** 1 Department of Plant Pathology and Microbiology, Bioenvironmental Sciences, The College of Agriculture and Life Sciences, Texas A&M University, Texas, USA

I have read this submission. I believe that I have an appropriate level of expertise to confirm that it is of an acceptable scientific standard.
